# The role of Octenidol^®^, Glandomed^®^ and chlorhexidine mouthwash in the prevention of mucositis and in the reduction of the oropharyngeal flora: a double-blind randomized controlled trial

**DOI:** 10.3205/dgkh000248

**Published:** 2015-02-13

**Authors:** Nico T. Mutters, Thomas R. Neubert, Rudolf Nieth, Reinier Mutters

**Affiliations:** 1 Heidelberg University Hospital, Department of Infectious Diseases, Heidelberg, Germany; 2 Marburg University Hospital, Wound and Pain Unit, Coordination Centre for Clinical Trials, Marburg, Germany; 3 Marburg University Hospital, Department of Haemato-Oncology, Marburg, Germany; 4 Marburg University Hospital, Institute for Medical Microbiology and Hygiene, Marburg, Germany

**Keywords:** mucositis, randomized controlled trial, oropharyngeal flora, octenidol, chlorhexidine, glandomed

## Abstract

**Aim:** The oropharyngeal flora is of importance for the development of oral mucositis, which is a frequent complication in oncologic practice. It also plays a role in the pathogenesis of ventilator-associated pneumonia. Mucositis is associated with significantly worse clinical and economic outcomes. The aim of our study was to assess the efficacy of Octenidol^®^, Glandomed^®^ and chlorhexidine mouthwash in the prevention of mucositis and reduction of the oropharyngeal flora.

**Methods:** A prospective, double-blinded RCT including two strata was conducted between October 2008 and November 2010. Stratum i consisted of ventilated cardiothoracic surgical patients. Stratum ii consisted of medical patients with haemato-oncological malignancies requiring stem cell transplantation. The primary outcome measures were development of mucositis regarding to OMAS/WHO score and reduction of the oropharyngeal flora.

**Results:** Both strata showed low OMAS/WHO scores which did not differ significantly between the groups. The overall mean reduction of colony forming units was significantly higher in the Octenidol^®^ group compared to the chlorhexidine and the Glandomed^®^ groups.

**Conclusions:** No significant differences in the development of mucositis were found, thus all solutions proved successful in the prevention of mucositis. However, Octenidol^®^ was superior in the reduction of the oropharyngeal flora. Hence, the preventive effect on nosocomial infections might be higher in patients using Octenidol^®^ rather than chlorhexidine or Glandomed^®^.

## Zusammenfassung

**Ziel:** Die oropharyngeale Flora ist in der Pathogenese der oralen Mukositis und der Beatmungs-assoziierten Pneumonie von großer Wichtigkeit. Die Mukositis als häufige Komplikation bei hämato-onkologischen Patienten, ist assoziiert mit signifikant verschlechtertem klinischen und ökonomischen Outcome. Das Ziel unserer Studie war die Überprüfung der Wirksamkeit von Octenidol^®^, Glandomed^®^ und Chlorhexidin-Mundspüllösung in Bezug auf die Prävention von Mukositis und die Reduktion der oropharyngealen Flora.

**Methoden:** Eine prospektive, doppelt-verblindete randomisierte Studie von Oktober 2008 bis November 2010 mit zwei Studienarmen. Arm i bestand aus beatmeten kardiochirurgischen Patienten. Arm ii bestand aus hämato-onkologischen Patienten, die eine Stammzelltransplantation erhielten. Primäre Endpunkte waren die Entwicklung von Mukositis nach dem OMAD/WHO Score sowie die Reduktion der oropharyngealen Flora.

**Ergebnisse:** Beide Studienarme zeigten niedrige OMAS/WHO Werte, die sich nicht signifikant unterschieden. Die gemittelte Gesamtreduktion von koloniebildenden Einheiten war signifikant größer in der Octenidol^®^-Gruppe im Vergleich zur Chlorhexidin- und Glandomed^®^-Gruppe.

**Fazit:** Es zeigten sich keine signifikanten Unterschiede in Bezug auf die Entwicklung von Mukositis in beiden Studienarmen. Alle Lösungen stellten eine erfolgreiche Präventionsmaßnahme in Bezug auf die Pathogenese von Mukositis dar. Octenidol^®^ zeigte im Vergleich zu Chlorhexidin oder Glandomed^®^ eine stärkere Reduktion der oropharyngealen Flora und könnte sich daher als effektiver in der Prävention nosokomialer Infektionen erweisen.

## Introduction

The oral microflora is of importance for the development of nosocomial infections, especially of ventilator-associated pneumonia (VAP) [1], [2], [3]. VAP is among the most frequently occurring nosocomial infections, particularly in critical ill patients [3], [4], [5]. Almost half of all nosocomial infections of patients in the intensive-care setting are VAPs [6]. Disruption of the regular oropharyngeal flora and colonisation with potentially pathogenic microorganisms is pivotal in the pathogenesis of VAP and of oral mucositis [1], [2], [3], [7], [8]. Topical oral decontamination can lead to a decrease of VAP incidence [3], [9]. However, there are also studies suggesting that oral decontamination has no significant effect on VAP incidence, albeit that those studies used chlorhexidine only [10]. A recent publication on the prevention of VAP stated, however, that selective decontamination of the oropharynx to decrease the microbial burden of the aerodigestive tract has a high quality of evidence and can be recommended [11]. 

Oral mucositis is a frequent complication of the chemotherapy and radiotherapy regimens commonly used in oncologic practice [8]. It is an especially severe problem for patients who are undergoing hematopoietic stem-cell transplantation [8]. Furthermore, oral mucositis is associated with significantly worse clinical and economic outcomes [8]. In the presence of neutropenia, severe mucositis also may predispose patients to septicaemia [8], [12], [13], [14]. In addition, from the patient’s perspective, oral mucositis is one of transplantation’s most debilitating side effects [15], [16].

The primary goals of our study were to assess the efficacy of octenidol compared to chlorhexidine and to glandomed in the reduction of the oropharyngeal flora and in the prevention of mucositis in ventilated surgical patients and in patients with haemato-oncological malignancies requiring stem cell transplantation.

## Material and methods

### Trial design

A prospective, double-blinded, randomized-controlled clinical trial including two strata was conducted at the University Hospital Marburg, Germany between October 1, 2008 and November 30, 2010. The trial protocol was approved by the institutional medical ethics committees. Written informed consent was obtained from all participants. The surgical stratum i consisted of ventilated cardiothoracic surgical patients and ventilated surgical patients regardless of a specific diagnosis. The haemato-oncological stratum ii consisted of medical patients with haemato-oncological malignancies requiring stem cell transplantation. For randomisation in both strata a computer-generated randomisation schedule was used to assign patients to the intervention group or to the control group. Stratum i received either Octenidol^®^ (Schülke & Mayr GmbH, Norderstedt, Germany) (intervention group) or chlorhexidine (control group). Stratum ii received either Octenidol^®^ (intervention group) or Glandomed^®^ (STADA, Bad Vilbel, Germany) (control group). Chlorhexidine was not used in stratum ii, since it is suggested that chlorhexidine mouthwash not be used in patients receiving radiation therapy and due to inadequate and/or conflicting evidence in patients receiving chemotherapy [17], [18].

Glandomed^®^ was included in our study design to have an additional control group, since it is a biologically inactive mouthwash. Glandomed^®^ contains macrogol (polyethylene glycol, PEG) but has no antiseptic properties and is used for moistening the oral mucosa in order to prevent mucositis. Glandomed^®^, however, contains a small amount (<0.1%) of chlorhexidine as adjuvant, which is added as a chemical stabilizer. In stratum i microbiological data were collected at baseline (first day of ventilation, T0), and at day 3 (T1) and 7 (T2). In stratum ii microbiological data collection was done at baseline (before chemotherapy was started, T0) and at day 3 (T1) and 7 (T2). Microbiological samples were taken in stratum i and stratum ii at T0, T1, T2. In addition, grade of mucositis was assessed using the Oral Mucositis Assessment Scale (OMAS) and the WHO mucositis scale [8], [19], [20]. 

### Patient inclusion

Between October 1, 2008 and November 30, 2010, all patients older than 18 years who were admitted to a surgical ICU or cardiosurgical ICU and were mechanically ventilated for ≥24 h independently of a specific diagnosis (stratum i) and all patients older than 18 years who were admitted to the haemato-oncology ward to receive a myeloablative allogeneic or autologous hematopoietic stem cell transplantation after high dose chemotherapy were eligible for the trial. For inclusion in the trial, the written informed consent form needed to be signed and personally dated by the patient or by the patient’s legally acceptable representative if the patient was already ventilated, and by the person who conducted the informed consent discussion. Exclusion criteria included absence of written informed consent; infections of the oropharyngeal region; dental defects; infections of the upper respiratory tract; mental disorders; hypersensitivity to chlorhexidine, glandomed or octenidine; temperature of >38°C before administration of chemotherapy (only stratum ii).

### Interventions

In stratum i a 0.1% chlorhexidine solution was used in the control group and a 0.1% octenidine-dihydrochlorid (Octenidol^®^) solution was used in the intervention group. Both solutions were used as an oral rinse (100 ml). In addition, 50 ccm per flushing (4 times daily) of chlorhexidine or Octenidol^®^, respectively, were applied to buccal, pharyngeal, gingival and tooth surfaces for 30 seconds.

In stratum ii a Glandomed^®^ solution was used in the control group and a 0.1% octenidine-dihydrochlorid solution was used in the intervention group. Both solutions were used as an oral rinse (100 ml), as well. In addition, 50 ccm per flushing (4 times daily) of Glandomed^®^ or Octenidol^®^, respectively, were applied to buccal, pharyngeal, gingival and tooth surfaces for 30 seconds. Subsequently, suction was applied to eliminate remaining liquid. Microbiological samples were taken at the above mentioned times of the collection of the data. Grade of mucositis was assessed by trained study nurses under control of the supervisor.

### End points and definitions

Culture results were provided by the department of medical microbiology, University of Marburg, Germany. Medical records of all patients were reviewed. The primary outcome measures were reduction of mucositis regarding to OMAS or WHO and reduction of the oropharyngeal flora. The secondary endpoints assessed grade and distribution of oral colonization with aerobic, microaerophilic and anaerobic gram-positive and gram-negative microorganisms.

### Microbiological methods

Samples (patient’s swabs from buccal, pharyngeal, gingival and tooth surfaces, and 2 ml of saliva) were inoculated on Columbia 5% sheep blood (COS) agar plate and chocolate agar plate (Becton Dickinson, Heidelberg, Germany); then incubated for 48 h under aerobic conditions at 36°C. For anaerobes samples were inoculated additionally on COS and selective Schaedler KV (supplemented with vitamin K1, 5% sheep blood and with kanamycin and vancomycin) and incubated for 72 h under anaerobic conditions at 36°C. Broth cultures were conducted with CASO-bouillon (Heipha, Dr. Mueller GmbH, Eppelheim, Germany) supplemented with LTHTh (inactivators lecithin, Tween 80, histidine and thiosulfate). Identification of bacterial growth and differentiating of periopathogens was performed with standard biochemical tests (catalase etc.), commercial identification systems (Crystal anaerobes, BD Heidelberg, Germany; Walkaway, DADE-Siemens, Marburg, Germany) and especially by matrix-assisted laser desorption ionization-time-of-flight mass spectrometry (Bruker, Billerica, MA, USA), as described elsewhere [21]. The number of colony forming units (CFU) was assessed by placing the swab in a vessel containing 5 mL of a minerals basic solution (5 mL) and serial dilutions were prepared, finally 0.1 mL samples were plated out on Columbia blood agar plates. The same procedure was accomplished with the rinsing solution. The agar plates were incubated aerobically and anaerobically. Each sample was incubated at 36°C for 48 h.

### Statistics

To detect a relevant reduction of the OMAS or WHO score rate of 20%, with a confidence level of 5% and a power of 80%, a minimum of 45 patients per stratum were required. Anticipating a dropout rate of approximately 5%, we planned to include 60 patients per stratum. Statistical analysis was performed using Statistical Package for the Social Sciences (SPSS 19.0, Chicago, USA). Descriptive statistics were used to explore data. Categorical and continuous variables were analyzed using either Student *t* tests or nonparametric tests, where appropriate. A two-sided *P* value less than 0.05 was considered significant. Normal distribution was tested with Kolmogorov-Smirnov-test. The Spearman rank-order correlation coefficient (*r*) was calculated to characterize correlation strength between the WHO mucositis score and the OMAS score. 

## Results

### Patient characteristics

**Figure 1:**
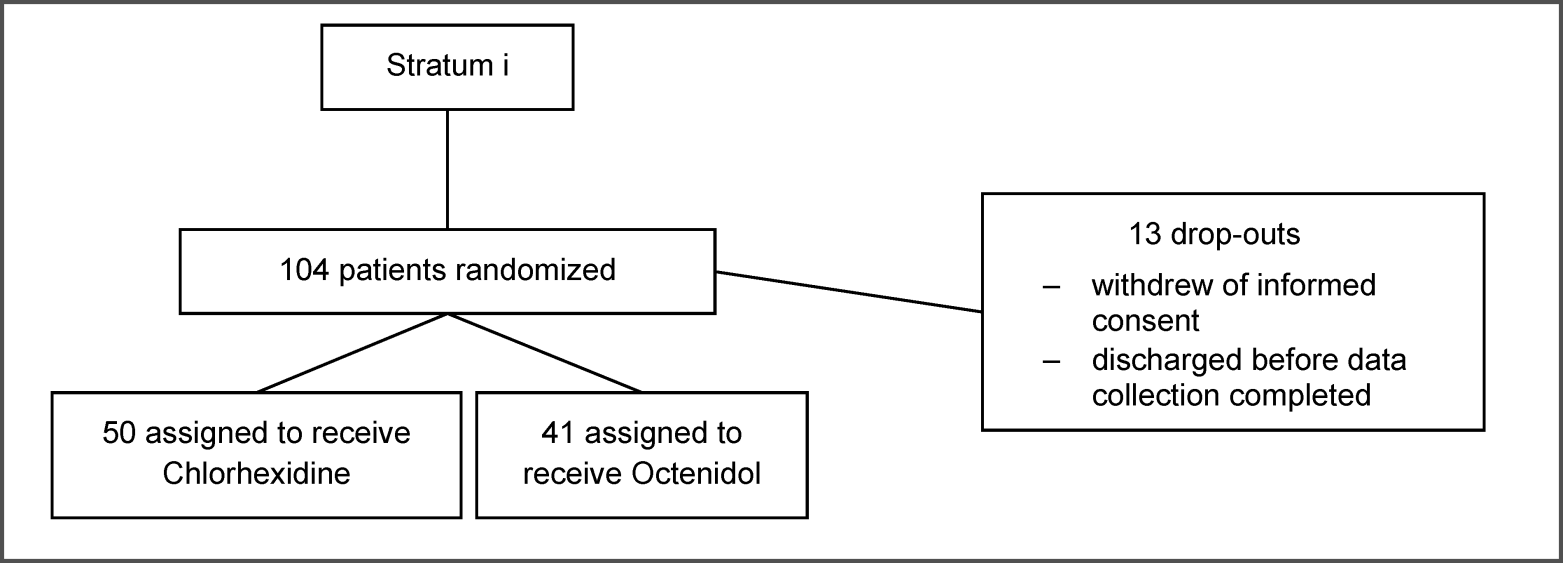
Flow of patients, stratum i surgical

**Figure 2:**
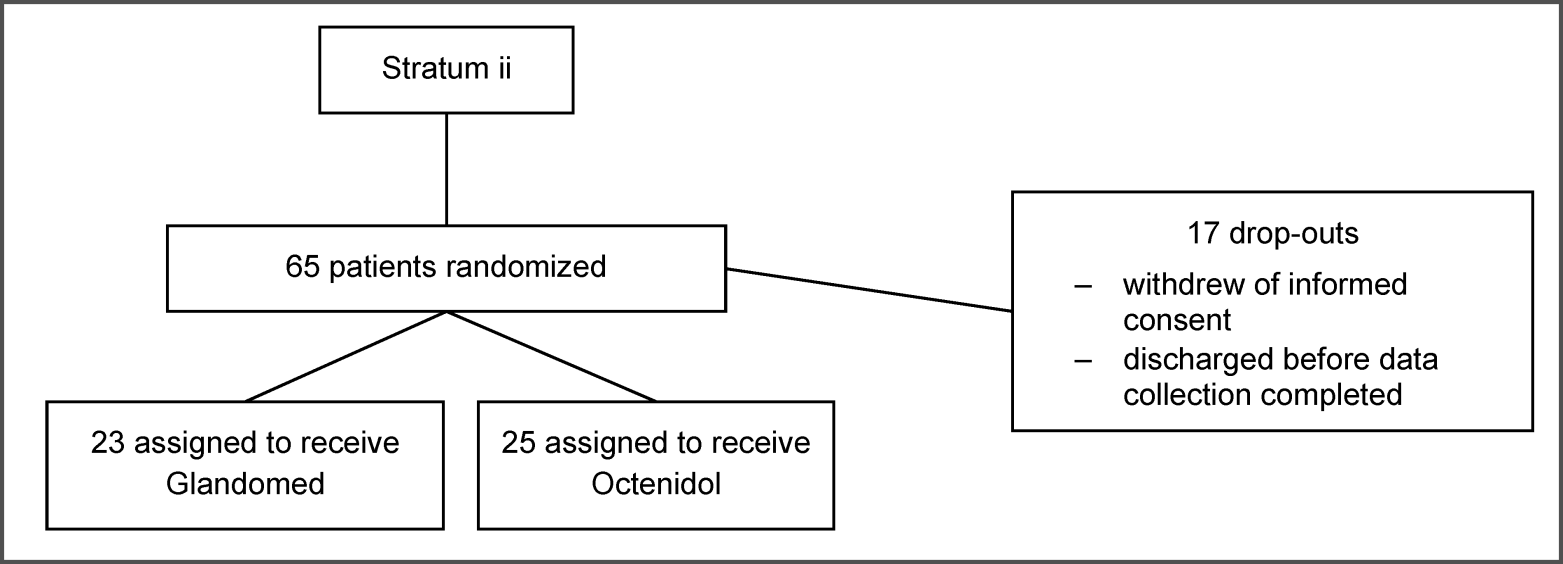
Flow of patients, stratum ii haemato-oncology

Patient characteristics are shown in Table 1 (Tab. 1). The flow of participants of the two strata is shown in Figure 1 (Fig. 1) and Figure 2 (Fig. 2).

### OMAS and WHO mucositis score

A high correlation between the two scores were found with a spearman correlation coefficient (*r*) for all measurement times T1, T2, T3 of 0.92, 0.93, 0.95, respectively (p=0.01) in the haemato-oncology stratum.

In stratum i and stratum ii, both groups showed low OMAS scores which did not differ significantly between the chlorhexidine, the glandomed or the octenidol group during all measurements times (Table 1 (Tab. 1)). In addition, the WHO scores were in the mean below grade 2 in all groups and differences were not significant between the two groups, as well (Table 1 (Tab. 1)). 

### Microbiological results

Stratum i showed a mean reduction of overall CFU of 4.7 log (SD 9.0) from T0 to T1, and of 4.5 log (SD 9.2) from T1 to T2, respectively. The Octenidol group showed a mean overall reduction of CFU at T1 of 7.1 log (SD 9.2) and of 7.6 log (SD 10.4) at T2, respectively, while the chlorhexidine group showed a reduction of only 2.6 log (SD 8.4) at T1 and of 2.3 (SD 7.3) at T2, respectively. The differences were statistically significant for T2 (p≤0.05), however not for T1 (p=0.08).

Stratum ii showed a mean reduction of overall CFU of 1.0 log (SD 4.6) at T1, and of 1.5 log (SD 5.7) at T2, respectively. The Octenidol^®^ group showed a mean overall reduction of CFU at T1 of 1.7 log (SD 5.0) and of 3.4 log (SD 5.8) at T2, respectively, while the Glandomed^®^ group showed a reduction of only 0.4 log (SD 4.4) at T1 and of 0.1 (SD 5.3) at T2, respectively. The differences were statistically significant for T2 (p=0.02), however not for T1 (p=0.27).

The overall mean reduction of CFU for stratum i and stratum ii compiled, was higher in the Octenidol^®^ group compared to the chlorhexidine or the Glandomed^®^ group (Table 2 (Tab. 2)). The differences were statistically significant for both T1 (p=0.04) and T2 (p=0.003). The reduction of CFU depicted per bacteria type (aerobic, microaerophilic and anaerobic) are shown in Table 3 (Tab. 3).

## Discussion

In our study, octenidol proved to be clearly superior in the reduction of the oropharyngeal flora compared to chlorhexidine or glandomed while no significant differences in the development of mucositis were found. Compared to chlorhexidine, octenidol has been shown to be less cytotoxic [22]. In addition, octenidol shows higher *in vitro* reduction rates of periodontal testorganisms compared to chlorhexidine [23].

Previous studies have shown heterogenic results in regards to the development of mucositis in patients using chlorhexidine. Some studies showed that usage of chlorhexidine can lead to an elevated mucositis score [24], [25], while Ferretti et al. showed that chlorhexidine mouthrinse significantly reduced the incidence of oral mucositis [26]. However, in our study we did not see a significant change in mucositis scores neither for the chlorhexidine nor for the octenidol or the glandomed group. In all groups the mucositis scores were low during the whole study period. Especially in patients undergoing chemo- and radiotherapy mucositis is a frequent complication and is associated with significantly worse clinical and economic outcomes [8]. Our results affirmed that all solutions, octenidol, chlorhexidine, and glandomed mouthwash effectively prevent the development of mucositis. The reduction of the oropharyngeal flora was significantly higher in the octenidol group. Thus, the preventive effect on nosocomial infections might be higher in patients using octenidol than patients using chlorhexidine or glandomed alone. One drawback of our study is that we did not assess the incidence rates of nosocomial infections in both groups, since we focused on the outcome mucositis only. However, other studies have shown that 1 extra nosocomial infection can be prevented if 16 patients are decontaminated [27]. The estimated cost to prevent 1 nosocomial infection was calculated as € 192 or US $ 230, only [27]. Selective oral decontamination has been shown to cost only US $ 100 for 8 days of treatment per patient and therefore being an extremely cost-effective strategy [3]. In addition, the usage of antiseptics is preferable compared to the topical application of antibiotics, since it avoids the potential increase in the selection of antibiotic-resistant pathogens.

Our study confirmed that the usage of antiseptic mouthwashes is effective in the prevention of mucositis, However, since initial mucositis rates were low in both strata, a prolonged study length might be necessary to confirm if the prevention effects will be present in the long-term, as well. Both Octenidol^®^ and chlorhexidine proved efficient in preventing mucositis of haemato-oncological and ventilated cardiothoracic patients. Interestingly, usage of a non-antiseptic solution alone, such as Glandomed^®^, appears to be as effective in the prevention of mucositis as usage of antiseptic solutions. Nevertheless, Glandomed^®^ contains a small amount (<0.1%) of chlorhexidine as adjuvant. Although chlorhexidine in such low concentrations should not have an antiseptic effect, it cannot be excluded that even such low concentrations of chlorhexidine might have biased the results of the control group. Administering sterile water only to the control group, however, cannot be conducted due to obvious ethical reasons. Octenidol^®^ was clearly superior in the reduction of the oropharyngeal flora compared to chlorhexidine and Glandomed^®^ and may be the preferable choice, since the preventive effect on nosocomial infections might be higher.

## Notes

### Meeting presentation

Parts of the sections material and methods and results have been presented in a poster on the 65^th^ annual meeting of the DGHM (Deutsche Gesellschaft für Hygiene und Mikrobiologie) in Rostock, September 2013.

### Competing interests

The authors declare that they have no competing interests.

### Funding

The study was funded by a grant from Schülke & Mayr.

## Figures and Tables

**Table 1:**
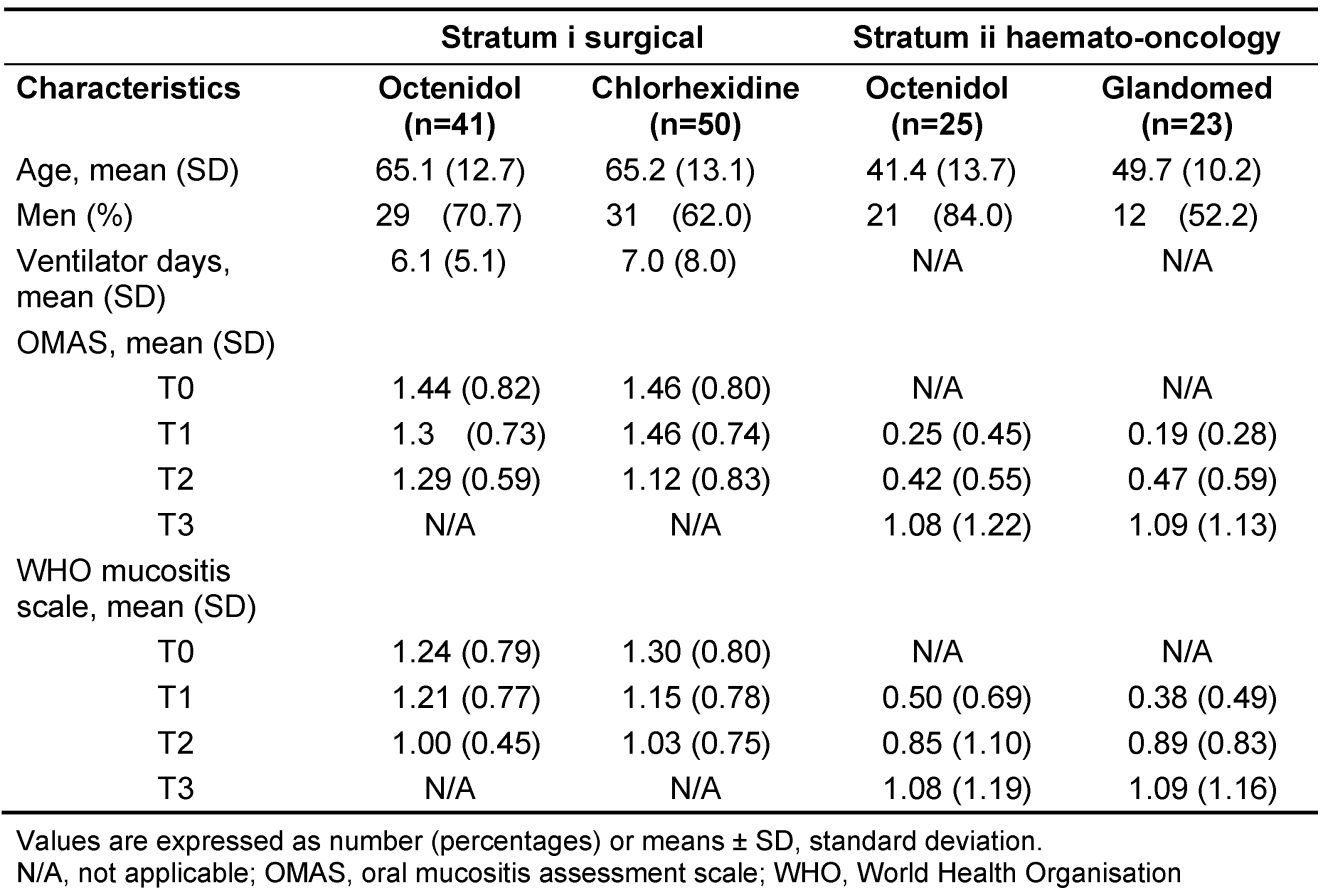
Patient characteristics and score results

**Table 2:**
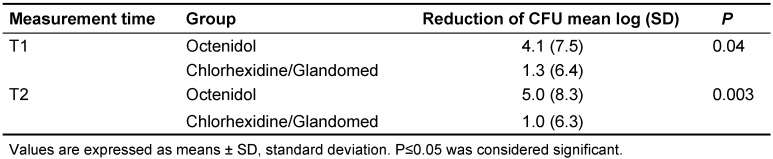
Overall reduction of CFU in both strata compiled

**Table 3:**
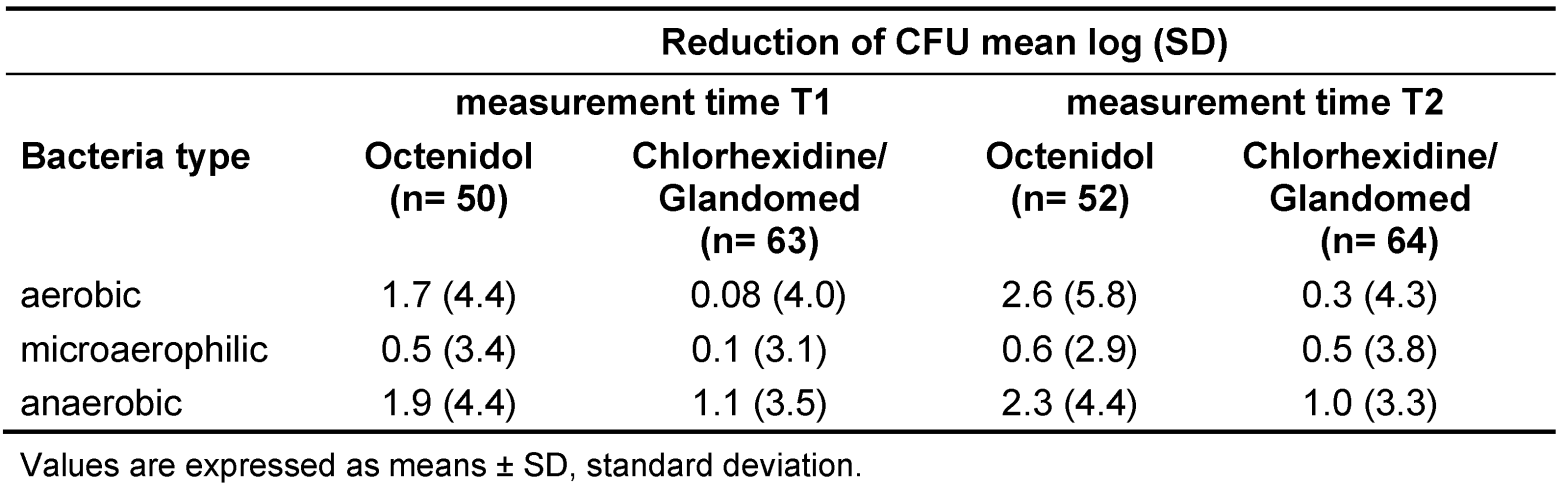
Overall reduction of CFU in both strata compiled depicted per bacteria type

## References

[1] Johanson WG, Pierce AK, Sanford JP, Thomas GD (1972). Nosocomial respiratory infections with gram-negative bacilli. The significance of colonization of the respiratory tract. Ann Intern Med.

[2] Bonten MJ, Gaillard CA, de Leeuw PW, Stobberingh EE (1997). Role of colonization of the upper intestinal tract in the pathogenesis of ventilator-associated pneumonia. Clin Infect Dis.

[3] Koeman M, van der Ven AJ, Hak E, Joore HC, Kaasjager K, de Smet AG, Ramsay G, Dormans TP, Aarts LP, de Bel EE, Hustinx WN, van der Tweel I, Hoepelman, AM, Bonten MJ (2006). Oral Decontamination with Chlorhexidine Reduces the Incidence of Ventilator-associated Pneumonia. Am J Respir Crit Care Med.

[4] Schwab F, Meyer E, Geffers C, Gastmeier P (2012). Understaffing, overcrowding, inappropriate nurse:ventilated patient ratio and nosocomial infections: which parameter is the best reflection of deficits?. J Hosp Infect.

[5] Tamayo E, Álvarez FJ, Martínez-Rafael B, Bustamante J, Bermejo-Martin JF, Fierro I, Eiros JM, Castrodeza J, Heredia M, Gómez-Herreras JI, Valladolid Sepsis Study Group (2012). Ventilator-associated pneumonia is an important risk factor for mortality after major cardiac surgery. J Crit Care.

[6] Vincent JL, Bihari DJ, Suter PM, Bruining HA, White J, Nicolas-Chanoin MH, Wolff M, Spencer RC, Hemmer M (1995). The prevalence of nosocomial infection in intensive care units in Europe. Results of the European Prevalence of Infection in Intensive Care (EPIC) Study. EPIC International Advisory Committee. JAMA.

[7] Johanson WG, Pierce AK, Sanford JP (1969). Changing pharyngeal bacterial flora of hospitalized patients. Emergence of gram-negative bacilli. N Engl J Med.

[8] Sonis ST, Oster G, Fuchs H, Bellm L, Bradford WZ, Edelsberg J, Hayden V, Eilers J, Epstein JB, LeVeque FG, Miller C, Peterson DE, Schubert MM, Spijkervet FK, Horowitz M (2001). Oral mucositis and the clinical and economic outcomes of hematopoietic stem-cell transplantation. J Clin Oncol.

[9] Fourrier F, Cau-Pottier E, Boutigny H, Roussel-Delvallez M, Jourdain M, Chopin C (2000). Effects of dental plaque antiseptic decontamination on bacterial colonization and nosocomial infections in critically ill patients. Intensive Care Med.

[10] Pineda LA, Saliba RG, El Solh AA (2006). Effect of oral decontamination with chlorhexidine on the incidence of nosocomial pneumonia: a meta-analysis. Crit Care.

[11] Klompas M, Branson R, Eichenwald EC, Greene LR, Howell MD, Lee G, Magill SS, Maragakis LL, Priebe GP, Speck K, Yokoe DS, Berenholtz SM (2014). Strategies to prevent ventilator-associated pneumonia in acute care hospitals: 2014 update. Infect Control Hosp Epidemiol.

[12] Classen DC, Burke JP, Ford CD, Evershed S, Aloia MR, Wilfahrt JK, Elliott JA (1990). Streptococcus mitis sepsis in bone marrow transplant patients receiving oral antimicrobial prophylaxis. Am J Med.

[13] Elting LS, Bodey GP, Keefe BH (1992). Septicemia and shock syndrome due to viridans streptococci: a case-control study of predisposing factors. Clin Infect Dis.

[14] Ruescher TJ, Sodeifi A, Scrivani SJ, Kaban LB, Sonis ST (1998). The impact of mucositis on alpha-hemolytic streptococcal infection in patients undergoing autologous bone marrow transplantation for hematologic malignancies. Cancer.

[15] Rubenstein EB, Peterson DE, Schubert M, Keefe D, McGuire D, Epstein J, Elting LS, Fox PC, Cooksley C, Sonis ST, Mucositis Study Section of the Multinational Association for Supportive Care in Cancer, International Society for Oral Oncology (2004). Clinical practice guidelines for the prevention and treatment of cancer therapy-induced oral and gastrointestinal mucositis. Cancer.

[16] Bellm LA, Epstein JB, Rose-Ped A, Martin P, Fuchs HJ (2000). Patient reports of complications of bone marrow transplantation. Support Care Cancer.

[17] Lalla RV, Bowen J, Barasch A, Elting L, Epstein J, Keefe DM, McGuire DB, Migliorati C, Nicolatou-Galitis O, Peterson DE, Raber-Durlacher JE, Sonis ST, Elad S, Mucositis Guidelines Leadership Group of the Multinational Association of Supportive Care in Cancer and International Society of Oral Oncology (MASCC/ISOO) (2014). MASCC/ISOO clinical practice guidelines for the management of mucositis secondary to cancer therapy. Cancer.

[18] McGuire DB, Fulton JS, Park J, Brown CG, Correa ME, Eilers J, Elad S, Gibson F, Oberle-Edwards LK, Bowen J, Lalla RV, Mucositis Study Group of the Multinational Association of Supportive Care in Cancer/International Society of Oral Oncology (MASCC/ISOO) (2013). Systematic review of basic oral care for the management of oral mucositis in cancer patients. Support Care Cancer.

[19] Sonis ST, Eilers JP, Epstein JB, LeVeque FG, Liggett WH, Mulagha MT, Peterson DE, Rose AH, Schubert MM, Spijkervet FK, Wittes JP (1999). Validation of a new scoring system for the assessment of clinical trial research of oral mucositis induced by radiation or chemotherapy. Mucositis Study Group. Cancer.

[20] Scully C, Epstein J, Sonis S (2004). Oral mucositis: a challenging complication of radiotherapy, chemotherapy, and radiochemotherapy. Part 2: diagnosis and management of mucositis. Head Neck.

[21] Eigner U, Holfelder M, Oberdorfer K, Betz-Wild U, Bertsch D, Fahr AM (2009). Performance of a matrix-assisted laser desorption ionization-time-of-flight mass spectrometry system for the identification of bacterial isolates in the clinical routine laboratory. Clin Lab.

[22] Kramer A, Müller G (2007). Mikrobiozide Wirksamkeit, weitere biologische Wirkungen, Verträglichkeit und Abbaubarkeit von Octenidindihydrochlorid. GMS Krankenhaushyg Interdiszip.

[23] Mutters R, Bykow H, Kulhat M (2007). Mikrobiozide Wirksamkeit antiseptischer Mundspüllösungen auf Basis von Octenidin, Chlorhexidin bzw. Amin-/Zinnfluorid gegenüber Parodontitis-Erregern. GMS Krankenhaushyg Interdiszip.

[24] Pitten FA, Kiefer T, Buth C, Doelken G, Kramer A (2003). Do cancer patients with chemotherapy-induced leukopenia benefit from an antiseptic chlorhexidine-based oral rinse? A double-blind, block-randomized, controlled study. J Hosp Infect.

[25] Foote RL, Loprinzi CL, Frank AR, O'Fallon JR, Gulavita S, Tewfik HH, Ryan MA, Earle JM, Novotny P (1994). Randomized trial of a chlorhexidine mouthwash for alleviation of radiation-induced mucositis. J Clin Oncol.

[26] Ferretti GA, Raybould TP, Brown AT, Macdonald JS, Greenwood M, Maruyama Y, Geil J, Lillich TT, Ash RC (1990). Chlorhexidine prophylaxis for chemotherapy- and radiotherapy-induced stomatitis: a randomized double-blind trial. Oral Surg Oral Med Oral Pathol.

[27] Segers P, Speekenbrink RG, Ubbink DT, van Ogtrop ML, de Mol BA (2006). Prevention of nosocomial infection in cardiac surgery by decontamination of the nasopharynx and oropharynx with chlorhexidine gluconate: a randomized controlled trial. JAMA.

